# Falciparum Malaria in Japan: Diagnostic Difficulty in Non-endemic Areas and the Importance of Immediate Referral

**DOI:** 10.7759/cureus.78568

**Published:** 2025-02-05

**Authors:** Mitsunobu Toyosaki, Mao Tsukadaira, Yushi Matsuo, Junichi Sasaki

**Affiliations:** 1 Department of Emergency and Critical Care Medicine, Keio University School of Medicine, Tokyo, JPN; 2 Department of Emergency Medicine, Fussa Hospital, Fussa, JPN

**Keywords:** case report, diagnostic challenges, emergency medicine, falciparum malaria, immediate referral, imported malaria, malaria diagnosis, non-endemic areas, travel malaria

## Abstract

In Japan, malaria is rare, and only a few institutions have the diagnostic capabilities to treat it. In falciparum malaria, the symptoms and signs of the disease are non-specific, and diagnostic delay in symptomatic patients can be fatal. Herein, we describe a case of falciparum malaria that achieved good outcomes in a patient who initially visited an institution in Japan that lacked malaria diagnostic resources. A female patient in her twenties was admitted to the emergency department with a high fever. She developed a fever three days after returning to Japan from Guinea, a malaria-endemic region. Additionally, she exhibited impaired consciousness, tachycardia, and tachypnea. Her laboratory results indicated an increased inflammatory response, thrombocytopenia, hyperbilirubinemia, and coagulopathy. Computed tomography revealed hepatic enlargement and splenomegaly. Based on these findings, malaria was suspected, and the patient was immediately referred to an infection-specialized hospital. There, the patient was diagnosed with falciparum malaria and was treated and discharged without increased severity. This case highlights the importance of promptly referring patients suspected of malaria who are returning from endemic areas to institutions with relevant diagnostic resources if required resources are unavailable.

## Introduction

Although malaria is a common disease in many countries (including most African regions and a part of Southeast Asia and Eastern Mediterranean regions), it is rare and non-endemic in Japan. In Japan, all malaria cases reported in the past 60 years have been imported, while locally transmitted cases were last observed in the Ryukyu Islands over six decades ago [1]. Approximately 50 to 100 imported malaria cases are reported in Japan each year [2]. However, increasing temperatures could potentially make Japan susceptible to malaria outbreaks in the future.

There are five malaria species that can be transmitted between humans: *Plasmodium falciparum, Plasmodium vivax, Plasmodium ovale curtisi, Plasmodium ovale wallikeri, and Plasmodium malariae*. Falciparum malaria has recently accounted for 70-80% of imported malaria cases in Japan and can progress rapidly to severe forms, which can be fatal without treatment [3]. Therefore, early diagnosis and effective treatment within 24-48 hours of symptom onset are essential [4]. However, the symptoms, signs, or laboratory data of malaria cases are non-specific, and a definitive diagnosis is generally made via light microscopy or rapid diagnostic tests at well-equipped institutions. In non-endemic regions like Japan, only a limited number of institutions have the diagnostic capability and experienced personnel for malaria. For example, in Tokyo, Japan's largest city, there are approximately 640 hospitals and 14,000 clinics [5], yet fewer than 20 institutions can diagnose and treat malaria. Herein, we present a case of a patient with falciparum malaria who achieved good outcomes after initially visiting an institution lacking malaria diagnostic resources in Japan.

## Case presentation

A female patient from Guinea in her twenties, residing in Japan, presented to the emergency department of our hospital with a primary complaint of a high fever. Three days after returning to Japan from Guinea, a malaria-endemic area, she developed fever, headache, and nausea. The patient had no significant medical history. Her vital signs upon arrival were as follows: consciousness assessed by Glasgow Coma Scale, with a score of 11 (E3V3M5), heart rate of 138 bpm, blood pressure of 131/82 mmHg, respiratory rate of 41/minute, oxygen saturation of 100% on room air, body temperature of 39.4℃, and pupils remain unexamined. Laboratory data revealed white blood cell count within the normal range (6100/µL), increased inflammatory response (C-reactive protein, 19.81 mg/dL), and decreased platelet count (53000/µL). The values of the following hepatobiliary enzyme levels were: aspartate aminotransferase, 17 U/L; alanine transaminase, 17 U/L; lactate dehydrogenase, 373 U/L; and bilirubin blood test, 1.63 mg/dL. Coagulopathy was also noted, with an activated partial thromboplastin time of 35.8 s, prothrombin time (PT) of 77.1% and 15.1 s, and PT-international normalized ratio of 1.18 (Table 1).

**Table 1:** Laboratory data at emergency department. CRP: C-reactive protein; Cl: chloride; Ca: calcium; Na: sodium; K: potassium; AST: aspartate aminotransferase; ALT: alanine transaminase; LDH: lactate dehydrogenase; BUN: blood urea nitrogen, CRE: creatinine; PT: prothrombin time; APTT: activated partial thromboplastin time; INR: international normalized ratio.

Variables	Examination findings	Normal range
Total protein (g/dl)	6.2	6.7-8.3
Albumin (g/dl)	2.8	3.9-4.9
Total-bilirubin (mg/dl)	1.63	0.20-1.20
AST (U/l)	17	8-38
ALT (U/l)	17	4-44
LDH (U/l)	373	106-211
Glucose (mg/dl)	154	60-110
BUN (mg/dl)	7.8	5.0-20.0
CRE (mg/dl)	0.57	0.50-1.30
Na (mmol/l)	128.7	135-147
K (mmol/l)	3.09	3.5-5.5
Cl (mmol/l)	96.6	98-108
Ca (mg/dl)	7.1	8.8-10.2
Inorganic P (mg/dl)	1	2.5-4.5
CRP (mg/dl)	19.81	0-0.3
White blood cell (10^2^/µl)	61	35-80
Red blood cell (10^4^/µl)	423	370-500
Hemoglobin (g/dl)	10.8	11.3-15.0
Hematocrit (%)	31.7	33.0-44.0
Platelet (10^4^/µl)	5.3	15.0-35.0
APTT (seconds)	35.8	25.0-40.0
PT (%)	77.1	70.0-130.0
PT sec (seconds)	15.1	-
PT-INR	1.18	0.90-1.10
Fibrinogen (mg/dl)	712	150.0-400.0

**Figure 1:**
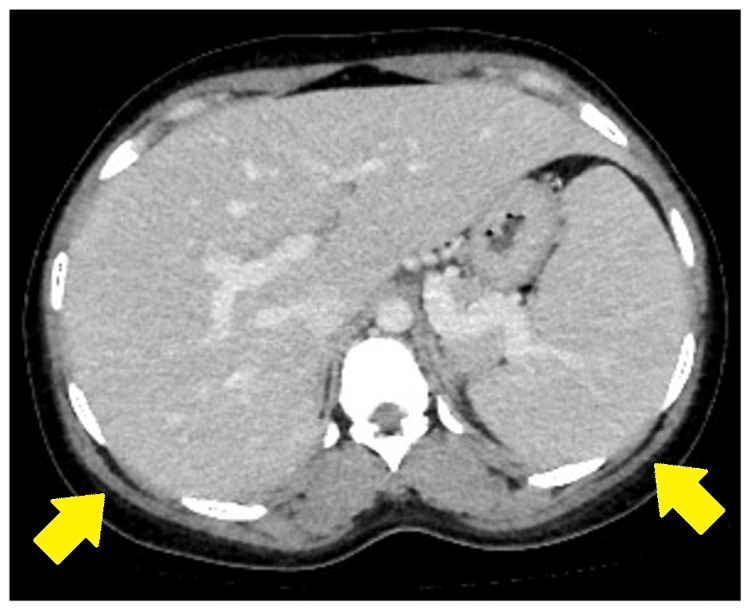
Hepatic enlargement and splenomegaly on contrast-enhanced computed tomography scan.

Coronavirus disease and influenza antigen tests were performed, and the results were negative. Contrast-enhanced computed tomography (CT) was implemented to search for the focus point of the infection or inflammation. Hepatic enlargement and splenomegaly were confirmed via CT (Figure 1).

**Figure 2:**
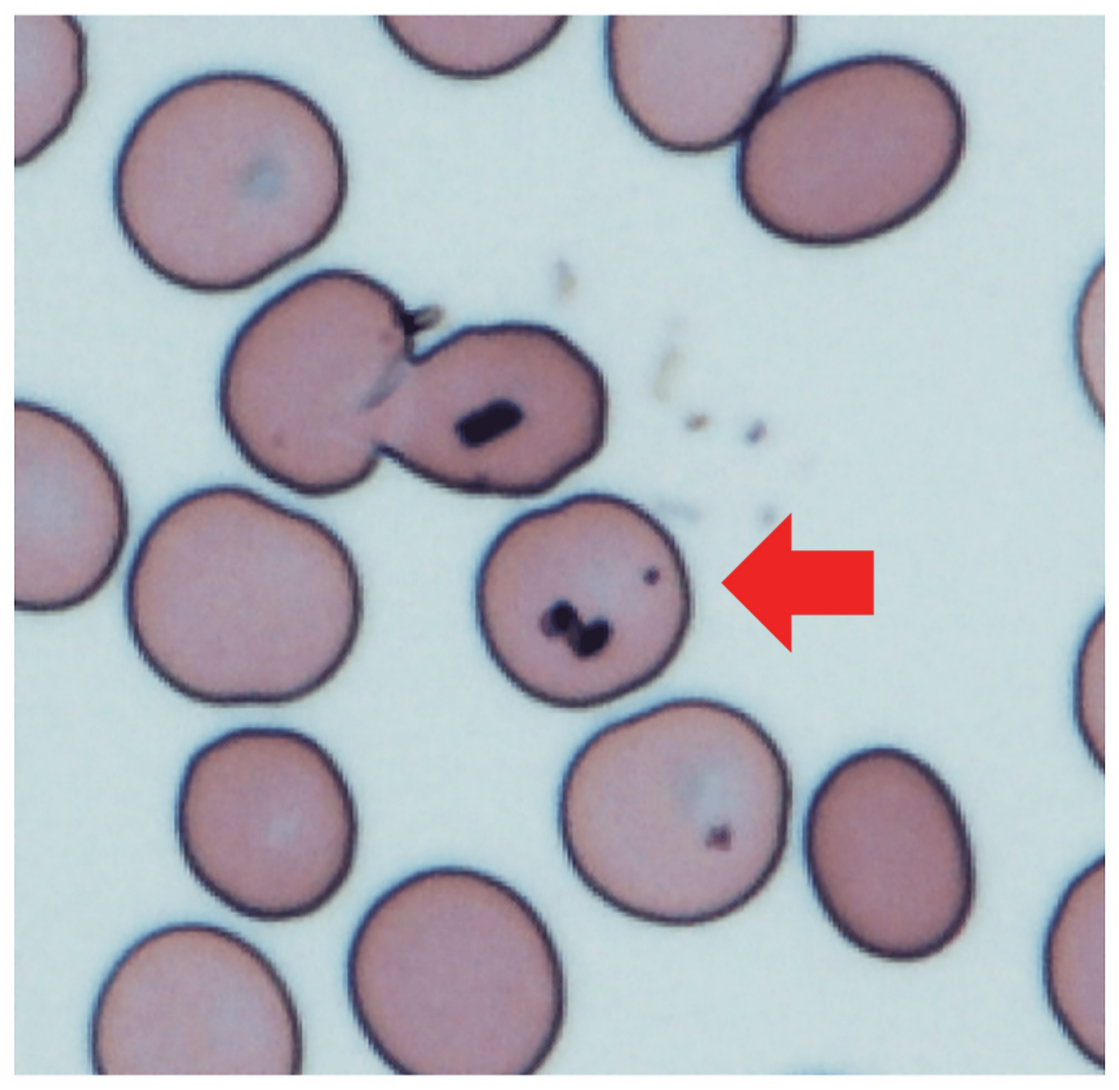
Malarial parasites on Giemsa-stained blood smears (×40).

Giemsa-stained blood smears were done routinely to observe blood cells. In this case, malformed red blood cells were confirmed. Based on the patient’s travel history, symptoms, laboratory data, and CT findings, malaria was considered one of the differential diagnoses. However, the parasites were not seen in the Giemsa-stained blood smears. The patient was immediately referred to an infection-specialized hospital, where malaria could be diagnosed and treated. At the referral hospital, the patient was diagnosed with falciparum malaria, and a parasitemia density of 0.08% was confirmed. In addition, we reviewed the same sample after hearing the diagnosis of malaria from the infection-specialized hospital, where we referred the patient. Parasites were confirmed in our blood smears after a more rigorous examination (Figure 2).

After oral administration of artemether-lumefantrine (artemether 20 mg and lumefantrine 120 mg) two tablets twice daily, she was discharged on the fifth day without an increase in symptom severity.

## Discussion

Diagnostic difficulty in institutions lacking malaria diagnostic resources and experience

Giemsa-stained blood smears, the gold standard of malaria definitive diagnosis, can be performed in most institutions lacking malaria diagnostic resources and were performed in our case before referral. However, the parasites could not be detected despite the clinical laboratory technician’s experience in diagnosing malaria parasites. Later, the institution to which the patient was referred confirmed a parasitemia density of 0.08%. Although malaria diagnosis by light microscopy has been the standard of definitive diagnosis for a long time, its accuracy is heavily dependent on the examiners' skills and parasitemia densities. Under typical field conditions, the limit of light microscope sensitivity is approximately 100 parasites/µL (0.002%) [6]. In our case, at an institution lacking malaria diagnostic resources in a non-endemic area, the diagnostic ability of light microscopy did not meet this standard. This indicated the diagnostic difficulty at an institution lacking specialized diagnostic capacity even when using a simple diagnostic method, such as light microscopy. Additionally, rapid diagnostic tests, which are another standard for malaria diagnosis, are unavailable in most institutions in Japan.

Challenges in diagnostic delay: determining the appropriate timing for referring patients to specialized institutions with diagnostic capacity

Diagnostic delays of malaria have often been reported in Japan [7-9]. In many of these cases, first (and subsequent) clinicians failed to consider the probability of malaria and instead diagnosed the patients with the common cold [7] or prescribed antibacterial drugs without a definitive diagnosis [8]. In Japan, the number of malaria cases is officially reported based on infectious disease laws, although mortality has not been officially reported. Old questionnaire surveys (1990-2000) reported a mortality rate of 3.3% in falciparum malaria cases [10]. On the other hand, in the U.S. (also a non-endemic area), seven fatal cases among 1823 cases (including 251 severe cases) were reported in 2018 [11]. The seven patients who died were infected with falciparum malaria. Moreover, all of them experienced a delay in the diagnosis of or proper treatment for malaria.

To prevent diagnostic delays and improve outcomes of malaria, early referrals are required. In Tokyo, 10-15 imported malaria cases are reported annually [12]. Moreover, only 15 institutions can diagnose malaria, of which 12 can treat malaria [13]. Table 2 shows the list of institutions/hospitals in Japan that can treat malaria.

**Table 2:** List of institutions which can diagnose or treat malaria. Table is the authors original work. * clinic for outpatients only, ** pediatric specialty hospital.

Institutions that can diagnose malaria	Malaria treatment
Self-defense Forces Central Hospital	Yes
The Jikei University Katsushika Medical Center	No
Toranomon Hospital	Inquiry required
Tokyo Takanawa Hospital	Inquiry required
Research Hospital, Institute of Medical Science, University of Tokyo	Yes
The Jikei University Hospital	Yes
Central Hospital of the National Center for Global Health and Medicine	Yes
Tokyo Metropolitan Ebara Hospital	Yes
Tokyo Metropolitan Toshima Hospital	Yes
Tokyo Metropolitan Cancer and Infectious Diseases Center, Komagome Hospital	Yes
St. Luke's International Hospital	Yes
Tokyo Medical University Hospital	Yes
Global Healthcare Clinic*Tokyo	Yes
Tokyo Metropolitan Tama Medical Center	Yes
Tokyo Metropolitan Children's Medical Center**	Yes

According to a U.S. systematic review, the presence of fever, splenomegaly, hyperbilirubinemia, or thrombocytopenia makes malaria more likely to recur in travelers. Therefore, laboratory testing is required for diagnosis [14]. In our case, the presence of all four of the abovementioned symptoms and signs sufficiently suggested the probability of malaria before referral. Although antimalarial treatment can be administered in settings without parasitological diagnostic capabilities [4], the low frequency of use has resulted in most institutions in Japan lacking malaria diagnostic resources and antimalarial drugs. In Japan, the universal health insurance system is in place, prioritizing cost reduction, particularly for diagnosing and treating rare diseases like malaria. Therefore, it is difficult to acquire rapid diagnostic tests or stock antimalarial drugs in all hospitals. Immediate referral to specialized clinics is the best plan for patients suspected of malaria, as shown in the present case where the patient achieved a favorable outcome.

Even if symptoms, signs, or laboratory data indicate only a small potential of malaria, consultation when no causative agent has been established with the goal of eliminating rare diseases is important. Diagnostic delays can occur even at institutions with the capacity to diagnose and treat malaria, as seen during the coronavirus disease pandemic [9].

## Conclusions

We report a case of a patient diagnosed with falciparum malaria in Japan who initially visited an institution lacking malaria diagnostic resources. Owing to immediate referral, a good outcome was achieved. In cases of fever, splenomegaly, hyperbilirubinemia, or thrombocytopenia in travelers or people returning from malaria-endemic areas, malaria should always be considered as a differential diagnosis. Although this was a single case in Japan to the best of our knowledge, the experience required to diagnose and treat malaria is similarly insufficient in other non-endemic regions. Therefore, it is crucial to immediately refer cases to institutions with diagnostic capabilities to treat malaria patients in non-endemic areas.

## References

[1] (2024). Malaria in Japan (Article in Japanese). https://idsc.niid.go.jp/iasr/18/213/tpc213-j.html.

[2] (2024). Beware of malaria! (Article in Japanese). https://www.forth.go.jp/moreinfo/topics/useful_malaria.html.

[3] (2024). Infectious diseases weekly reports (Article in Japanese). Infectious Diseases Weekly Reports.

[4] WHO WHO (2024). WHO guidelines for malaria [Internet]. https://www.ncbi.nlm.nih.gov/books/NBK588130/.

[5] (2024). Medical institutions of Tokyo (Article in Japanese). https://www.metro.tokyo.lg.jp/tosei/hodohappyo/press/2022/11/14/documents/01_01.pdf.

[6] WHO WHO (1988). Malaria diagnosis: memorandum from a WHO Meeting. Bull World Health Organ.

[7] Hase R (2018). Diagnostic delay for imported malaria: a case of Plasmodium falciparum malaria misdiagnosed as common cold. J Gen Fam Med.

[8] Shibahara D, Kinjo T, Nishiyama N (2015). Falciparum malaria incidentally pretreated with azithromycin. Intern Med.

[9] Mito H, Hase R, Ueda H (2023). A pitfall of cognitive bias during the pandemic: Two cases of Plasmodium falciparum malaria coinfected or misdiagnosed with COVID-19. J Infect Chemother.

[10] (2024). Malaria prevention guidelines for Japanese travelers (Article in Japanese). https://jsparasitol.org/wp-content/uploads/2015/12/complete.pdf.

[11] Mace KE, Lucchi NW, Tan KR (2022). Malaria surveillance-United States, 2018. MMWR Surveill Summ.

[12] (2024). Epidemic situations of malaria in Tokyo (Article in Japanese). https://idsc.tmiph.metro.tokyo.lg.jp/diseases/malaria/malaria/.

[13] (2024). Post-travel hospital list (Article in Japanese). http://jstah.umin.jp/03posttravel/index.htm.

[14] Taylor SM, Molyneux ME, Simel DL, Meshnick SR, Juliano JJ (2010). Does this patient have malaria?. JAMA.

